# Ponseti Clubfoot Casting: Factors That Affect Trainee Competency (Retrospective Observational Study) 

**DOI:** 10.5435/JAAOSGlobal-D-22-00008

**Published:** 2022-02-15

**Authors:** Samuel O. Noonan, Scott Hetzel, Kenneth J. Noonan, John E. Herzenberg, Donald S. Bae, Benjamin J. Shore

**Affiliations:** From the University of Denver, Denver, Colorado (Noonan); the Department of Orthopaedic Surgery, Boston Children's Hospital, Harvard Medical School, Boston, MA (Dr. Bae and Dr. Shore); the Rubin Institute for Advanced Orthopedics, Sinai Hospital of Baltimore, Baltimore, MD (Dr. Herzenberg); and the University of Wisconsin School of Medicine and Public Health, Madison WI (Dr. Hetzel, Dr. Noonan).

## Abstract

**Introduction:**

This study investigates how previous simulation training and clinical experience affects trainee performance when manipulating a foot, applying a Ponseti clubfoot cast, and performing an Achilles tenotomy on a clubfoot simulator.

**Methods:**

Sixty-four Accreditation Council for Graduate Medical Education orthopaedic trainees participated in the 2017 to 2018 Top Gun (TG) skills competition at the International Pediatric Orthopaedic Symposium. Trainees were judged by expert pediatric orthopaedic surgeons on how they manipulated a clubfoot model, applied a cast, and performed a simulated tendoachilles tenotomy (TAT). An analysis was done to correlate the test variables with a contestant's TG Ponseti score.

**Results:**

Twenty-one contestants with previous residency training using synthetic clubfoot models scored higher (*P* = 0.007) than those trainees without training. Trainees who had applied >10 clubfoot casts and who participated in >10 TATs in training also scored higher (*P* = 0.038 and *P* = 0.01, respectively). Thirteen contestants who had previously attended an International Pediatric Orthopaedic Symposium meeting and seven contestants who attended a American Academy of Orthopaedic Surgery clubfoot workshop scored higher (*P* = 0.012 and *P* = 0.017 respectively).

**Discussion:**

Clinical and previous simulation experience related to the Ponseti method correlated with improved performance on our Ponseti simulation. Trainees who had previous experience with >10 clubfoot casts and >10 TATs scored higher during TG than less experienced trainees.

Historically, surgical education has followed a mentorship model of see one, do one, teach one.”^1^ While this teaching model has been effective since the early 1900s, increased pressure on operating room efficiency, combined with resident/trainee work-hour restrictions, has placed strain on the traditional apprenticeship model. The dictum of first do no harm is becoming harder to achieve.^2^ In response to these current educational challenges, simulation is being considered as an alternative and complementary means of resident education.^2,3,4,5^

Simulation training can be implemented on different learning platforms and may provide a safe and effective method of skill acquisition for all levels of trainees and practitioners.^6,7^ Innovative state-of-the-art simulation devices that train surgical skills, without risk to patients, allow for the detection and analysis of errors and near misses.^8,9^ In orthopaedics, a variety of surgical simulation platforms have been validated with early results to indicate improved trainee performance over time.^8,10,11,12,13,14,15,16,17,18,19,20^ Although surgical simulation/training opportunities relevant to pediatric orthopaedic procedures have demonstrated great promise and utility, more investigation is needed to identify duration and quality of knowledge and skill transfer.^21,22,23,24,25,26,27^

Pediatric clubfoot or congenital talipes equinovarus (CTEV) is one of the most common birth deformities.^28^ The three-dimensional pathoanatomy of a CTEV foot is complex and the sequential casting developed by Professor Ponseti requires hands on practice and repetition.^29^ Therefore, the management of CTEV and the application of clubfoot casts and tenotomy have been adopted as part of the Accreditation Council for Graduate Medical Education (ACGME) Pediatric Orthopaedic Fellowship Milestones as a core competency skill. Despite being a common birth deformity, trainees may complete their entire pediatric orthopaedic experience without treating a child with CTEV with the Ponseti method from presentation to onset of orthosis use. While the 80-hour workweek restrictions have decreased orthopaedic subspecialty rotations, gaining the skill of Ponseti casting and resident knowledge and performance by direct observed clinical practice can be challenging and even impossible.^30^

Casting and, particularly, Ponseti casting is a learned technique, and current literature highlights how different methods of training can improve trainee clubfoot cast application.^31,32,33,34^ The ability of a simulation program to assess the effectiveness of clubfoot cast application and tenotomy execution and relate that to a trainee's previous clinical experience has yet to be studied or reported in the literature. Therefore, the purpose of this study was to investigate how previous clinical and simulation experience affects trainee performance when applying a Ponseti clubfoot cast and performing an Achilles tenotomy on a clubfoot simulator. We hypothesize that there are certain variables that predict trainee competency in Ponseti clubfoot management (both casting and tenotomy). By studying the relationship between previous training and clinical experience on simulation performance, perhaps, we can identify certain thresholds for clinical competency around an important ACGME pediatric milestone and gather valued information for future studies analyzing the simulation clinical precision, validity, and effect.

## Methods

### Study Participants

**Figure 1:**
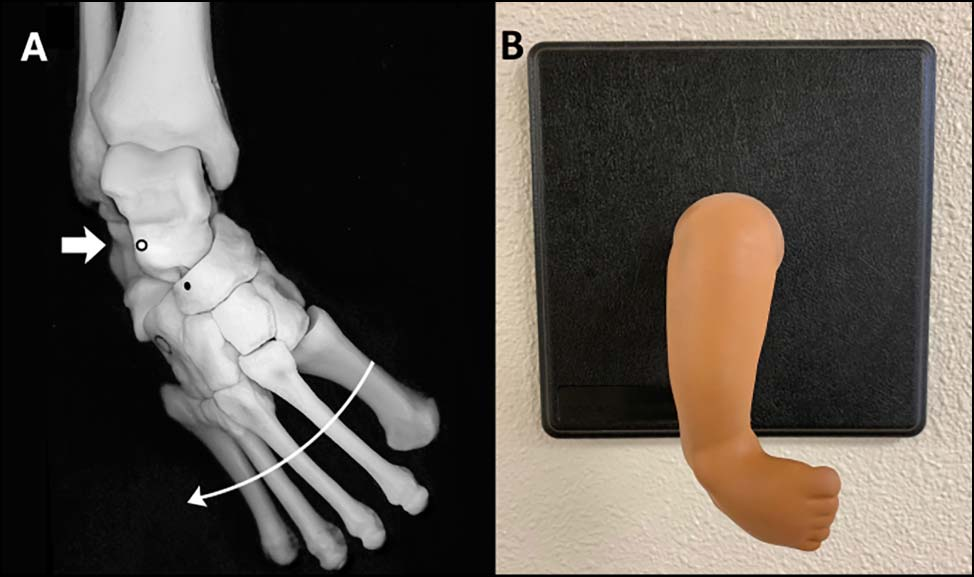
**A**, Radiograph showing anatomic bone model used to correlate the bony anatomy with soft tissue landmarks and to demonstrate manipulation. The forefoot is abducted (curved arrow) with a fulcrum at the talar head (short arrow). **B**, Photograph showing a right uncorrected clubfoot foot model used during the Ponseti casting portion of Top Gun. (MD Orthopaedics, Wayland, IA.)

**Figure 2:**
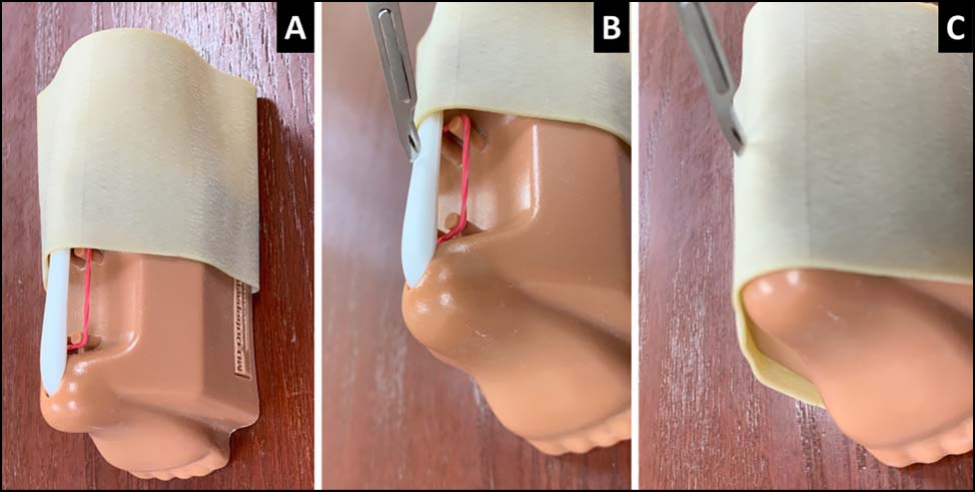
**A**, Photograph showing a Achilles tendon tenotomy model used during the Ponseti portion of Top Gun, with the rubber skin retracted and demonstrating the white Achilles tendon and adjacent neurovascular structures [red rubber band] (MD Orthopaedics, Wayland IA.) **B**, Photograph showing that the contestants must cut the white Achilles tendon and avoid the vessel. **C**, Photograph showing that with the tendon covered with rubber skin, a trainee cuts the Achilles.

In 2012, the American Academy of Orthopaedic Surgery (AAOS) and the Pediatric Orthopaedic Society of North America held the International Pediatric Orthopaedic Symposium (IPOS) where they introduced a surgical simulation contest, called Top Gun,” borrowed from the 1986 hit movie that depicted how the US Navy trained its top naval aviators. IPOS’ Top Gun (TG) was created to provide a fun, motor skills competition, focused on fundamental procedures related to pediatric orthopaedics, which ultimately highlighted the benefits of simulation training and fostered partnerships among stakeholders in orthopaedic education and patient care.^25^ TG has six core skills, one of which involved foot manipulation, application of Ponseti casting, and performance of Achilles tenotomy. In the clubfoot simulation, contestants demonstrated how to manipulate and perform the Ponseti casting sequence on a rubber clubfoot model (MD Orthopaedics) (Figure 1). In addition, contestants conducted a simulated tenotomy (TAT) on a hindfoot model (Figure 2) (MD Orthopaedics). Study participants consisted of residents and fellows participating in the TG portion of IPOS at the 2017 and 2018 meetings. While all trainees who participated received a score, this study only evaluated the scores and surveys from trainees who were participating in ACGME-accredited residency and fellowship training programs. Participants ranged from postgraduate year (PGY)3 to PGY7 experience. This study was a prospective analysis of trainee performance scores, combined with retrospective collected survey data related to Ponseti casting and the management of clubfoot.

### Intervention

Three weeks before TG, participants were required to review material for Ponseti clubfoot casting and performance of TAT. This included reading material and instructional videos. Participants were also given the scoring sheet ahead of time, which was used to grade the quality of their manipulation, clubfoot cast, and tenotomy (Appendix 1, http://links.lww.com/JG9/A195). Simultaneously, three weeks before TG, each competitor completed a precompetition survey that identified demographic details of the trainee (postgraduate year), level of educational experience in their training program (formal training on models, didactic lessons, etc.), and clinical experience with managing CTEV (number of casts applied and tenotomies done in their previous training). Applicants reported their comfort with the different aspects of the Ponseti casting method. Additional CTEV educational experiences such as participation in clubfoot seminars from the AAOS Annual Meeting and previous IPOS meetings were also recorded. During the competition, groups of six trainees rotated between the six skill stations for 15 minutes at each station, with no ability to compare notes or share what had transpired at the previous station to affect trainee performance and score. At the end of the Top-Gun Session, each participant filled out a postcompetition survey. This survey recorded whether they had attended a Ponseti workshop during the same IPOS meeting and before the TG competition and what factors could be done to improve the Ponseti simulation and educational experience.

The scoring methodology for the Ponseti station at TG was standardized during the study period by the Ponseti faculty. Six fellowship-trained pediatric orthopaedic surgeons observed and graded the applicants under the supervision of a captain. All judges had extensive experience in managing children with CTEV and the Ponseti method. Three judges were present for both 2017 and 2018, while three judges changed between the two years; the Captain was constant for both years. Each judge evaluated only one participant at a time. After the competition, the judges reconvened to address potential scoring concerns or questions to guarantee homogeneity in scoring.

The final scoring sheet was graded on a scale of 0 to 25 points, grouped into three sections that focused on landmarks/manipulation, cast application, or tenotomy (Appendix 1, http://links.lww.com/JG9/A195); no participants were awarded a perfect score. Participants were asked what landmarks should be used for the manipulation of a clubfoot and were instructed to identify where these landmarks were on the model. They were then told to apply a cast that would be an initial manipulation and casting, and to do this correctly, they would be required to know that cavus correction should be emphasized first with or without forefoot abduction. The purpose of Top-Gun was to test the skills required to correct a clubfoot with the Ponseti method (manipulation, casting, and TAT) rather than train the Ponseti method. During the TG session, trainees were observed, and the judges did not intervene when mistakes were made. At the completion of the session, a short debriefing occurred, which focused on strengths and weaknesses of a trainee's performance. This study was deemed IRB exempt by the senior author's IRB office. Statistical testing that analyzed previous experience on TG outcome included the Wilcoxon rank sum test and the Mann-Whitney *U* test. A *P* value less than 0.05 was considered significant.

## Results

A total of 72 trainees were available for analysis. Eight trainees were excluded because their training was from non–ACGME-accredited training programs, leaving 64 trainees with scores ranging from the 72nd to 91st percentile of a total score of 100. Most participants were in PGY3 and PGY4 (48/64, 75%) with limited previous clubfoot experience on models or patients (<5 casts [37/64, 58%]) (Table 1). Many applicants felt little to no competence with Ponseti casting before the simulation (43/64, 67%). Notably, attendance at an IPOS clubfoot workshop before TG did little to assuage the anxiety of the trainees who had attended. More than half of the applicants who indicated that they had participated in an IPOS Ponseti workshop before the TG competition (58.1%) were still uncomfortable.

**Table 1:** Top Gun Scores by Variable Training Parameters of North American Contestants

	N	TG Score	*P* value
Level of training			
PGY3	16	80.0 (72.0-81.0)	
PGY4	32	82.0 (76.0-85.0)	
PGY5-6	8	82.0 (80.0-89.0)	
Fellow	8	86.0 (84.0-88.0)	0.075
Level of training			
PGY3-4	48	80.0 (76.0-84.0)	
PGY5-6/Fellow	16	84.0 (80.0-88.0)	**0.045**
Previous training on rubber models			
No	43	80.0 (75.0-84.0)	
Yes	21	84.0 (80.0-88.0)	**0.007**
No. of clubfoot casts applied during training			
0	13	80.0 (76.0-88.0)	
<5	24	80.0 (76.0-85.0)	
5-10	21	80.0 (72.0-84.0)	
>10	6	88.0 (85.0-91.0)	**0.038**
No. of Achilles tenotomies attended in training			
<5	40	80.0 (76.0-84.0)	
5-10	16	80.0 (75.0-88.0)	
>10	8	88.0 (84.0-89.0)	**0.01**
“Do you feel competent in performing the Ponseti method?”			
No/little competence	43	80.0 (76.0-84.0)	
Moderate/very	21	84.0 (80.0-88.0)	**0.018**
Attended previous AAOS Ponseti Workshop			
No	57	80.0 (76.0-88.0)	
Yes	7	84.0 (84.0-90.0)	**0.017**
Attended previous IPOS meetings			
No	51	80.0 (75.0-84.0)	
Yes	13	84.0 (84.0-88.0)	**0.012**
Participate in an IPOS Ponseti			
No	26	80.0 (72.0-84.0)	
Workshop at any IPOS Before TG			
Yes	38	84.0 (77.0-88.0)	0.149

Bold = *p*<0.05 indicating statistical significance.

AAOS = American Academy of Orthopaedic Surgery, IPOS = International Pediatric Orthopaedic Symposium, TG = top gun

When contestants were stratified according to their level of training, we found that PGY5 residents and fellows obtained a higher performance score (*P* = 0.045). Previous experience with Ponseti models did translate into improved global scores (*P* = 0.007), indicating that overall performance is multifactorial and may be affected by trainee experience. Specifically, trainees who had previous clinical experience on actual patients performed better, with a threshold for greater than 10 clinical casts or greater than 10 TAT correlating with improved performance in the simulation (*P* = 0.038 and *P* = 0.01, respectively). Trainees demonstrated that they had accurate insight into their skill: those who felt moderately or very competent scored higher on the simulation (*P* = 0.018). Seven participants who previously attended an AAOS Annual Meeting workshop and 13 participants who previously attended an IPOS meeting achieved better global TG scores when compared with their inexperienced colleagues (*P* = 0.017) and (*P* = 0.012). Interestingly, no significant difference was observed in total scores seen in those contestants who attended the IPOS Ponseti hands-on workshop before TG competition.

## Discussion

Surgical skill development is an essential component of orthopaedic training but is at risk due to issues related to increasing cost, decreasing work hours, and risk to patient safety.^35^ Simulation provides a solution to the traditional model of apprenticeship skill development. In this study, we analyzed how previous clinical and simulation experience affected trainee performance associated with the Ponseti casting method when tested on a clubfoot simulator as judged by experts in the method. The goal of this study was to use a Ponseti simulator to test trainee competency based on their previous training experience (both clinical and model experience). In this study, we found that previous experience had a positive correlation with performance; specifically, trainees who had greater clinical experience (applied greater than 10 clubfoot casts and/or conducted greater than 10 Achilles tenotomies) achieved higher performance scores in our Ponseti simulation. Higher scores were seen in trainees with previous experience using the Ponseti simulation and in those applicants who were fellows and had additional years of training. The results presented here add credence to the statement that practice makes perfect, and in a sequenced procedure such as Ponseti clubfoot casting, the use of a simulator can be a reliable and valuable tool to assess trainee experience and performance.

The Ponseti treatment method for CTEV can be difficult to teach to inexperienced learners, given the nuances in appropriate pressure and molding associated with clubfoot casts. International clubfoot learning opportunities have developed to include the use of written materials, the Internet, Training the Trainer, e-Learning, and video learning which have improved the care of clubfoot.^31,32,36-38^ Using a clubfoot model to facilitate experience or to increase repetitions is of additional value for all levels of trainees.^38^ In this study, trainees with more experience using a Ponseti model and caring for children with CTEV performed better in cast application and tenotomy than inexperienced peers.

The use of surgical simulation as an adjunctive educational tool for the curriculum of orthopaedic surgical residents continues to grow in popularity. Simulation allows for iterative, deliberate, and problem-based learning and feedback without risk to real patients. Several studies have demonstrated the positive effect of trainee performance after simulation.^8,19,21,23,25,39,40^ Previous research at IPOS has demonstrated that a similar cohort of TG trainees improved knowledge and skill acquisition when exposed to a septic hip virtual simulator.^25^ Jackson et al.^27^ found that trainees exposed to a distal radius simulation model first performed better regarding casting and closed reduction of distal radius in the emergency department compared with a cohort who had no experience with simulation. Our study is different in that we did not prospectively compare two cohorts of clubfoot trainees (one with simulation exposure and one without). Our study looked at the outcome from a clubfoot competition when tested on simulation models and then looked at this outcome according to a retrospective review of previous trainee experience. Despite difference in study design, we experienced a similar phenomenon as Jackson et al.,^27^ where we found that clubfoot training with simulation models and previous experience can positively affect overall performance because it relates to simulated Ponseti clubfoot manipulation, casting, and Achilles tenotomy. Furthermore, a threshold of experience was needed to perform well with the Ponseti method, and perhaps, this number should be associated with ACGME milestones for the Ponseti method. In addition, we found that the current Ponseti simulator represents good construct validity because it mirrors what is being learnt clinically as those trainees with more experience achieved the highest scores on the simulator.

In this study, we sought to understand what factors correlate with trainee performance as it pertains to application of the Ponseti method for CTEV on a simulation model. We found that when trainees applied greater than 10 casts or were present for more than 10 Achilles tenotomies, we saw significantly higher performance. Furthermore, we found that trainees had accurate insight into their own skill proficiency. Those who felt they were confident in their Ponseti method scored higher than those who did not feel confident. Overall, we found that experience, either clinical or through simulation, was beneficial for trainee performance in the application of Ponseti casts and TAT for CTEV. As mentioned previously, participants who completed a hands-on Ponseti casting station at IPOS before the TG event did not perform better than those who did not. This was surprising, suggesting that skill transfer associated with Ponseti casting may be complex and multifactorial. During the IPOS Ponseti casting workshops, only 40% of the training was hands-on while most of the section was conducting through lecture. This may be an issue as Supramaniam et al^41^ demonstrated that hands-on simulation training is a superior training method compared with didactic lecturing on surgical skills. Our study's findings in association with the literature are being considered by the IPOS faculty as they continue to look to improve the IPOS casting workshop. Further research is needed to identify characteristics of knowledge transfer and duration of skill acquisition for trainees.

With growing restrictions on trainee's educational exposure, increasing strain has been placed on the quality and quantity of clinical learning opportunities. These restrictions have caused increasing concern among educators regarding trainee competence and confidence.^30^ In response, simulation training can effectively assess the competency and performance of trainees in a safe environment, with repeated measures.^24,42^ The results of our study demonstrated the potential of simulation to measure a trainee's proficiency in the ACGME pediatric milestone of Ponseti method for CTEV.

The results of our study should be interpreted cautiously based on the following limitations. We selected groups for testing the different variable in a manner that would allow enough power for analysis as noncontinuous variables. Alternatively, one could have analyzed some of these as continuous variables. While we tried to standardize our analysis of trainees, some variation was noted in our graders during the study period, which could have affected our results. Furthermore, quantifying the effect of a previous experience on a trainee is difficult when using a survey. Although many applicants indicated the amount of previous training and previous experience, the quality and detail of that experience is not unquantifiable in our surveys. We assume accurate recall by our trainees when completing the survey, but inaccuracies here could introduce recall bias, which could affect our conclusions. Our study was also not powered to differentiate casting performance among PGY level, and by dichotomizing trainee experience into two groups, we have limited the power of our analysis. We assessed performance of trainees in a timed, stressful scenario and tested only the initial manipulation and casting, and we acknowledge that additional factors may have affected performance, which were not controlled for in the study design. Another limitation of this study, which is true of any similar study of a simulation, is the question of how well it translates to actual treatment. Training and experience that improves one's ability to perform in a simulation may not translate directly to clinical expertise. From the data, we have identified 10 as the number of casts applied or tenotomies done as a threshold for competency, but we recognize that a larger sample of contestants may reveal a different threshold and identify other variables that potentially could affect competency. Despite these limitations, however, we believe important conclusions regarding simulation in general, and the utility of the Ponseti clubfoot simulation specifically, can be made.

In conclusion, previous clinical and simulation experience with a clubfoot model can positively affect trainee performance when manipulating a clubfoot, applying a cast, and performing an Achilles tenotomy in a CTEV simulation. The Ponseti method can be effectively taught to trainees, but this study shows that a minimum threshold is necessary for skill transfer and milestone performance. Our results suggest that a threshold of 10 Ponseti CTEV cases could be introduced into the milestone criteria for resident and fellow education. Because 75% of the contestants that had greater than 10 cast applications or tenotomies were pediatric orthopaedic fellows, this may imply that a fellowship is needed to gain enough experience for Ponseti casting competency. Further research into the duration and quality of knowledge transfer from simulation experiences is necessary to understand the ultimate education benefit for orthopaedic trainees.
